# Quantitative proteomics analysis of vitreous body from type 2 diabetic patients with proliferative diabetic retinopathy

**DOI:** 10.1186/s12886-018-0821-3

**Published:** 2018-06-26

**Authors:** Jianqing Li, Qianyi Lu, Peirong Lu

**Affiliations:** Department of Ophthalmology, the First Affiliated Hospital of Soochow University, No. 188 Shizi street, Suzhou, 215006 China

**Keywords:** Proliferative diabetic retinopathy, Quantitative proteomics, Bioinformatics

## Abstract

**Background:**

To compare the abundance of vitreous proteins between the patients with proliferative diabetic retinopathy (PDR) and idiopathic macular hole (IMH).

**Methods:**

In this study, we performed mass spectrometry-based label-free quantitative proteomics analysis of vitreous samples from type 2 diabetic patients with PDR (*n* = 9) and IMH subjects (*n* = 9) and identified the abundance of 610 proteins.

**Results:**

Out of 610 proteins, 64 proteins (Group A) were unique to PDR patients, while 212 proteins (Group B) could be identified in IMH vitreous only. Among the other 334 proteins that could be detected in both PDR and IMH eyes, 62 proteins differed significantly (*p* < 0.05, fold change > 2), which included 52 proteins (Group C) and 10 proteins (Group D) over- and under-expressed in PDR vitreous compared with the control. All proteins in these four groups were counted as significant proteins in our study.

**Conclusions:**

We identified and quantified 610 proteins in total, which included 338 significant proteins in our study. Protein distribution analysis demonstrated a clear separation of protein expression in PDR and IMH. The protein function analysis illustrated that immunity and transport related proteins might be associated with PDR.

## Background

Diabetic retinopathy (DR), a pathological condition in which damage occurs to the retina due to diabetes mellitus, is the most common microvascular complication of diabetes [1]. This disease can be further classified into two types: nonproliferative diabetic retinopathy (NPDR) and proliferative diabetic retinopathy (PDR). Classification of NPDR is based on clinical findings manifested by visible features, including microaneurysms, retinal hemorrhages, intraretinal microvascular abnormalities (IRMA), and venous caliber changes, while PDR is characterized by the hallmark feature of pathologic preretinal neovascularization [2]. With the increasing global prevalence of diabetes, DR is the major cause of vision loss and blindness among working-age adults in developed countries [3]. Current treatments for diabetic eye disease, which include laser photocoagulation, intravitreous injections of anti-VEGF and steroid agents and vitreoretinal surgery, mainly focus on advanced diseases such as PDR [4]. However, because of side effects and individual differences, there is an urgent need for new therapeutic options for PDR [5–7]. Therefore, a better understanding of the pathological mechanism is required for the development of new treatment options.

Research on PDR is limited because no current model can completely replicate the full pathophysiology of neuronal and vascular changes that occur at each stage of DR [8]. The physiologic and pathologic conditions of the retina are reflected in the protein composition of the vitreous due to their close anatomical and biological relationship, which can be sampled as part of routine surgical procedures [9, 10]. In this regard, vitreous fluid obtained through vitrectomy is currently used to indirectly examine the condition of the retina. Mass spectrometry (MS) has been widely applied to study biomolecules and one rapidly developing field is the global analysis of proteins, proteomics [11]. MS-based proteomics is of great value to study different ocular diseases such as cataracts [12], age-related macular degeneration (AMD) [13, 14] and DR [15, 16]. Despite the great efforts that have been devoted to DR vitreous protein identification [17, 18], we are still far from a comprehensive understanding of the complex molecular pathomechanisms. Quantitative proteomics is an analytical chemistry technique for determining the amount of proteins, which can contribute to better insight into the underlying pathogenesis [19].

In this study, the abundance of vitreous proteins was compared between the PDR and IMH subjects through a label-free quantitative method. We also analyzed protein distribution and then conducted bioinformatic analyses, which included protein function, related disease, gene ontology (GO), protein-protein interaction (PPI) and the Kyoto Encyclopedia of Genes and Genomes (KEGG) pathway.

## Methods

### Subject enrollment and sample collection

This prospective observational study was conducted at the First Affiliated Hospital of Soochow University according to the principles of the Declaration of Helsinki and the approved Human Discarded Specimen Research Protocols from the respective institutional review boards. The study was approved by the institutional ethics committee of the First Affiliated Hospital of Soochow University and signed informed consent was obtained from every participant before being included into the study. A total of 18 subjects who received vitrectomy were enrolled, 9 were type 2 diabetic patients with PDR, and 9 served as controls with IMH. The exclusion criteria were as follows: (1) acute or chronic infection, (2) vitreous hemorrhage or vitreous opacities, (3) a history of ocular surgery or laser photocoagulation, (4) other ocular diseases, (5) systemic diseases other than diabetes, and (6) use of antimetabolites or immunosuppressants.

The vitreous samples were collected by a syringe prior to the infusion procedure of the 3-port pars plana vitrectomy and then transferred into a centrifugal tube and stored at − 80 °C.

### Sample preparation

Vitreous samples were dissolved in reducing solution (6 M urea, 2 M thiourea; Sigma, St. Louis, MO) and then centrifuged at 12000 g for 45 min at 4 °C to collect the supernatant. Afterwards, the protein concentrations were measured by bicinchoninic acid (BCA) protein assay kit (Pierce, Thermo Scientific, Waltham, MA USA). For LC-MS analysis, 100 μg of protein from each sample was taken and then reduced with 10 mM dithiothreitol (Sigma, St. Louis, MO) at 37 °C for 2.5 h, alkylated with 50 mM iodoacetamide (Sigma, St. Louis, MO) at room temperature for 40 min followed by trypsin digestion with sequencing grade modified trypsin (Promega, Madison, WI, USA) using a 1:50 enzyme/protein ratio at 37 °C overnight. Tryptic peptides were purified with C18 microspin columns (Nest Group, Southborough, MA, USA).

### MS based label-free quantification

The purified peptide samples were loaded onto Orbitrap Elite hybrid mass spectrometer (Thermo Scientific) coupled to EASY-nLC II system (Thermo Scientific) using the Xcalibur version 2.7.0 SP1 (Thermo Scientific). The MS analysis was conducted in data-dependent acquisition where one high resolution (120000) FTMS full scan (m/z 300–1700) was followed by top20 CID-MS2 scans in ion trap (energy 35). Only the precursor ions with over 500 ion counts were allowed for MS^n^. Charge state rejection was enabled as well as dynamic exclusion which was fixed at 30 s for the selected ions.

The MS1 intensities of peptides for label-free quantification were acquired by the Progenesis LC-MS software (v 4.1, Nonlinear Dynamics Limited, Tyne, UK). For protein identification, the MS2-scan data obtained from Progenesis LC - MS were searched against the human component of the UniProtKB database using the SEQUEST search engine in Proteome Discoverer software (version 1.4, Thermo Scientific). The results were filtered to a maximum false discovery rate (FDR) of 0.05. Afterwards, spectral counts for each protein were extracted from the search results of the SEQUEST database and used to quantify protein abundance differences [20].

### Data processing

Student’s t test (*p* < 0.05) and fold change (fold change > 2) were calculated on protein abundance changes between PDR and IMH vitreous.

Analysis of protein distribution contained boxplot, correlation analysis and principal component analysis (PCA) which were produced using R version 3.4.1 and the hierarchical clustering analysis (HCA) of the identified proteins being constructed by Cluster 3.0.

DAVID bioinformatics Resources (Available online: https://david.ncifcrf.gov/) was employed to study significant proteins about protein functions, related disease and GO, which included biological processes, cellular components and molecular function.

PPI and KEGG pathway were analyzed through the String database (Available online: http://www.string-db.org) and produced by Cytoscape.

## Results

### Proteome

To analyze the differences in vitreous proteomics between PDR and IMH patients, we enrolled 9 subjects in each group. The difference of the protein concentration was not statistically significant in PDR vitreous and control eyes (5.23 ± 0.86 vs. 5.12 ± 0.56 μg/μl, *p* = 0.75). The proteins were then digested into peptides and analyzed by LC-MS/MS-based label-free quantitation.

We identified and quantified the abundance of 610 vitreous proteins. Among these, 64 proteins (Group A) could be identified only in PDR patients while 212 proteins (Group B) were unique to IMH vitreous. Among the other 334 proteins that could be detected in both PDR and IMH eyes, 62 proteins differed significantly (*p* < 0.05, fold change > 2), which included 52 (Group C) and 10 (Group D) that were up- and down- regulated, respectively, in PDR vitreous compared with the control. All proteins in these four groups were counted as significant proteins in our study.

### Protein distribution analysis

#### Boxplot

**Fig. 1:**
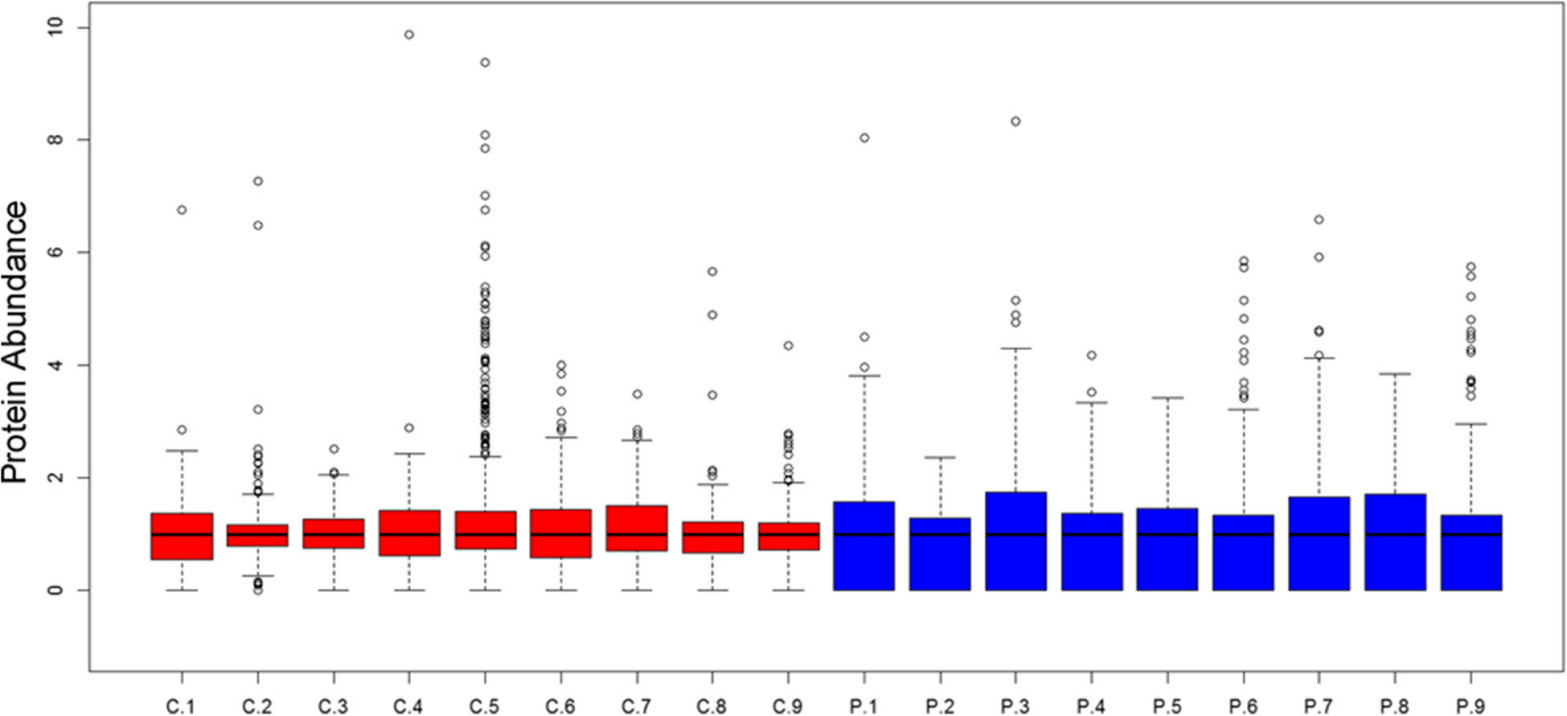
A boxplot produced by R version 3.4.1 about all proteins in each sample. C1 is short for the number 1 sample in the control group while P1 represents the number 1 sample in the proliferative diabetic retinopathy group. The results demonstrate a wider protein distribution in PDR samples than those in IMH vitreous. Furthers, more proteins in the control group have abnormal abundance and C5 is the most unreliable.

We made a boxplot pertaining to all of the proteins in the 18 samples. As shown in Fig. 1, the proteins in PDR samples had a wider distribution than those in IMH vitreous as the interquartile ranges and the distances between the upper limit and the lower limit were longer. In addition, the higher number of outliers in the control samples reflected more proteins with abnormal abundance than those in the PDR group. The number 5 sample in the control group (C5) had the most outliers that declared unreliability to some extent.

#### Correlation analysis

**Fig. 2:**
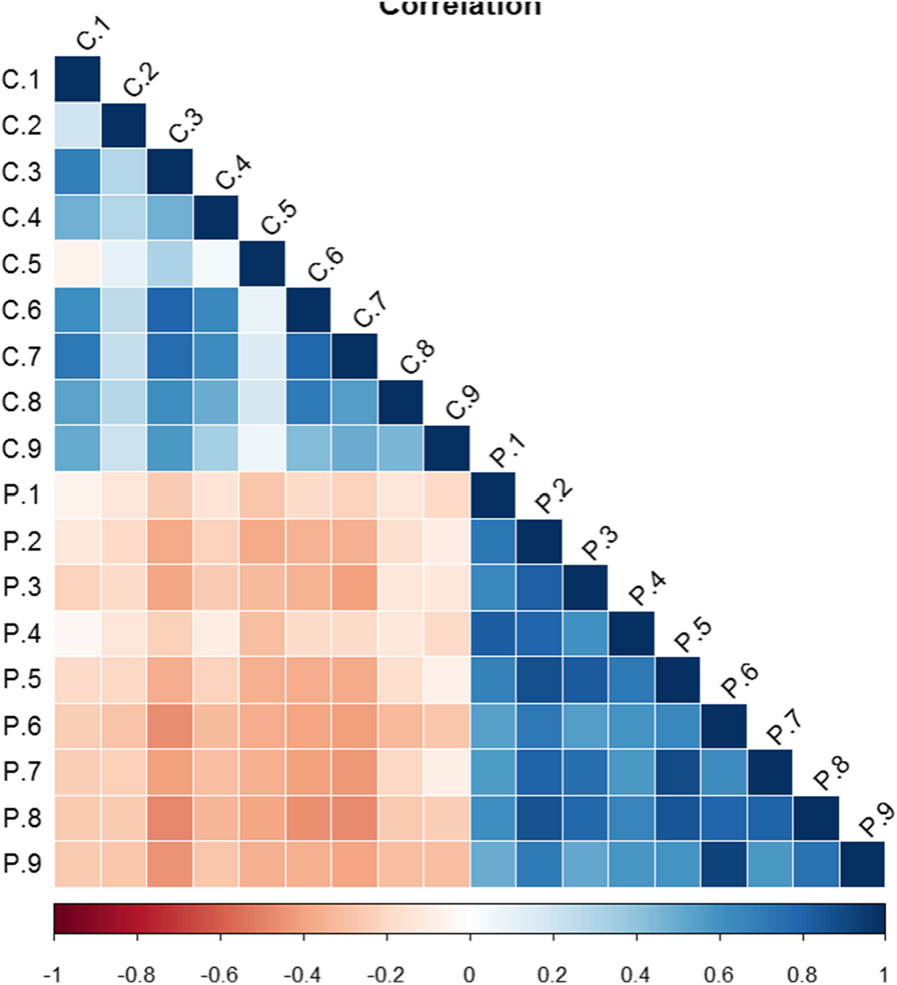
Correlation analysis of all proteins in every sample conducted by R version 3.4.1. The IMH samples have weaker correlation than PDR ones and C5 have the least correlation with the other IMH samples

Correlation analysis was conducted to confirm the distribution of our identified proteins. The value of correlation coefficient ranges from − 1 to 1. A positive value shows positive correlation, while a negative value is opposite. When the absolute value of correlation coefficient approaches 1, a stronger correlative extent exists, and when it approaches 0, a weaker correlative extent results. It is reflected in Fig. 2 that IMH samples indeed shared weaker correlations than those among PDR samples. In addition, C5 had the least correlation compared to the other IMH samples, which was in support of the conclusion from the boxplot.

**Fig. 3:**
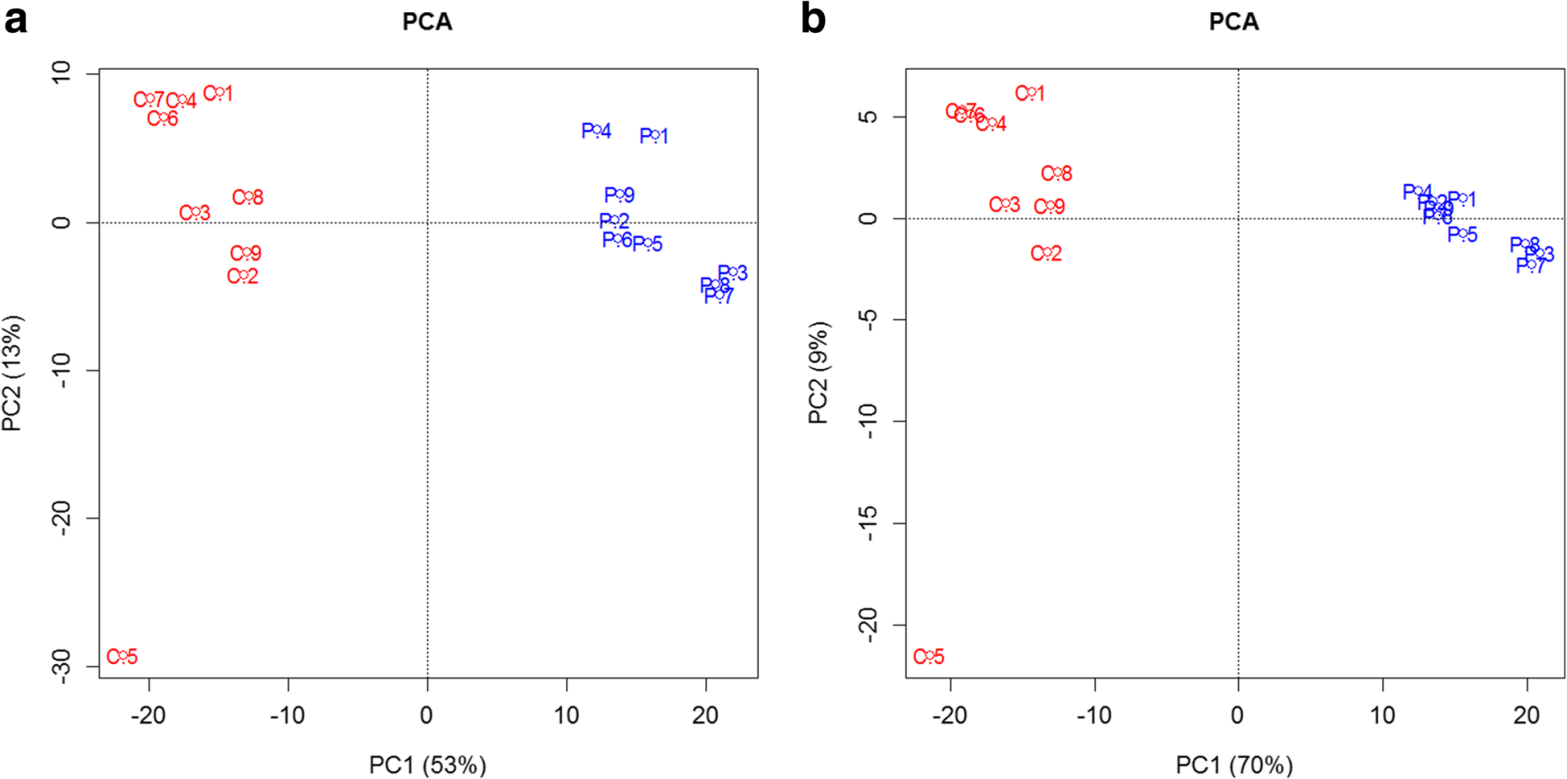
Principal component analysis (PCA) carried out by R version 3.4.1. **a** PCA of all proteins. **b** PCA of significant proteins. A clear difference in protein expression of the PDR group and the control group is displayed and C5 is proved to be unreliable again. The difference among the samples in one group becomes less when significant proteins been taken into account

**Fig. 4:**
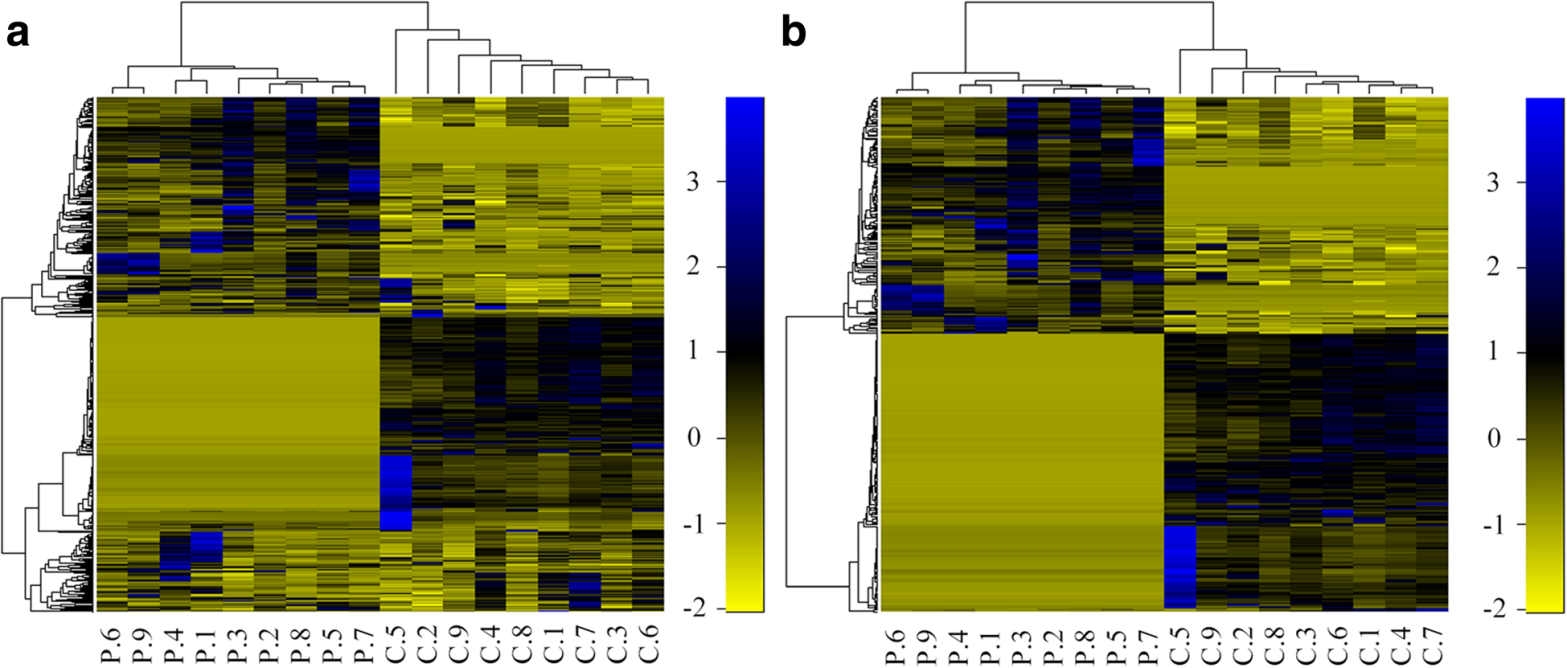
Hierarchical clustering analysis (HCA) by R version 3.4.1. **a** HCA of all proteins. **b** HCA of significant proteins. The color gradient illustrates the fold change of protein abundance between the two groups. A clear separation of the protein expression in the two disease states is shown and the separation becomes more apparent when significant proteins been taken into account

**Fig. 5:**
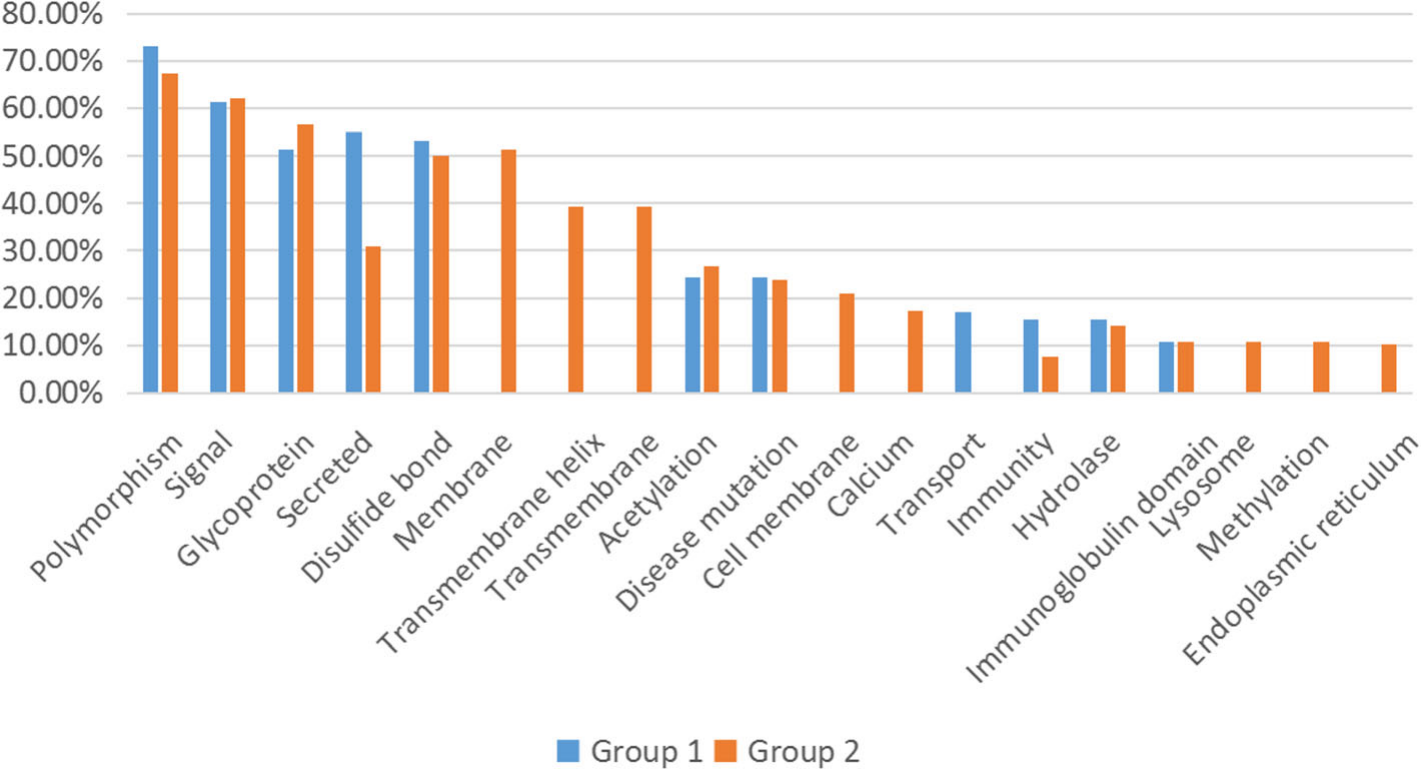
Protein functions detected by DAVID bioinformatics Resources relates to more than 10% proteins in two groups. Some elementary physiological functions are of high level in both groups. Furthers, immunity is associated significantly with more proteins in Group 1 than Group 2. Meanwhile, transport is unique to Group 1

**Fig. 6:**
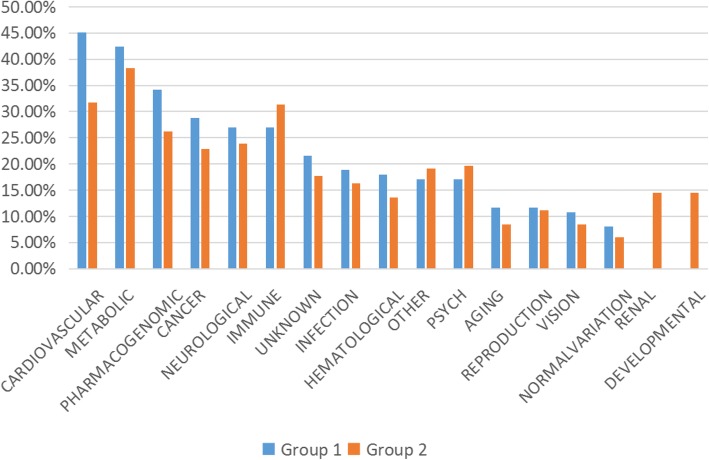
Related disease classification analyzed by DAVID bioinformatics Resources. It revealed that the top 6 disease categories were cardiovascular, metabolic, pharmacogenomic, cancer, neurological and immune

**Fig. 7:**
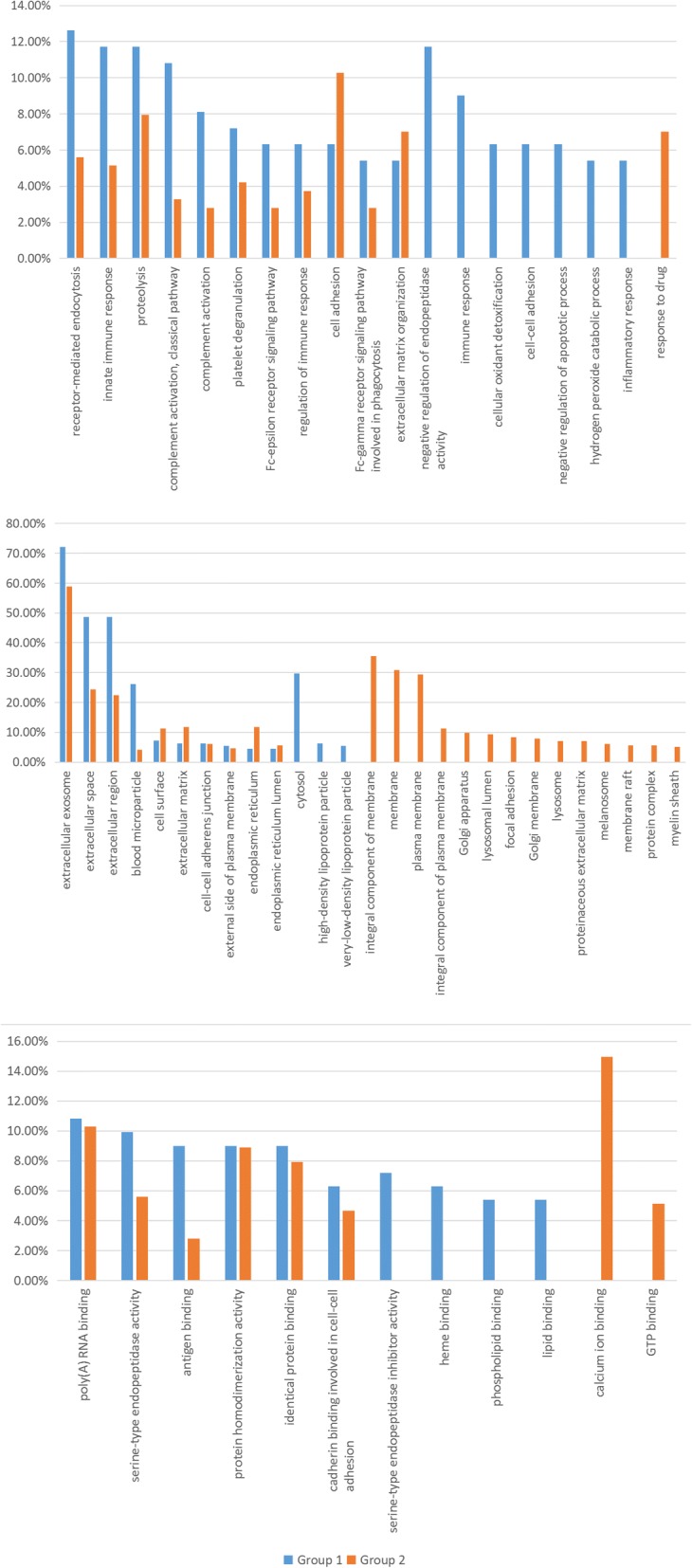
Gene Ontology which involves more than 5% significant proteins from the two groups. A. 5 biological processes relates significantly to more proteins in Group 1, and 7 biological processes are unique to Group 1. B. Three cellular components relate significantly to more proteins in Group 1, and 3 were unique to Group 1. C. Only 1 molecular functions relate significant to more proteins in Group 1, while 4 were unique to Group 1

**Fig. 8:**
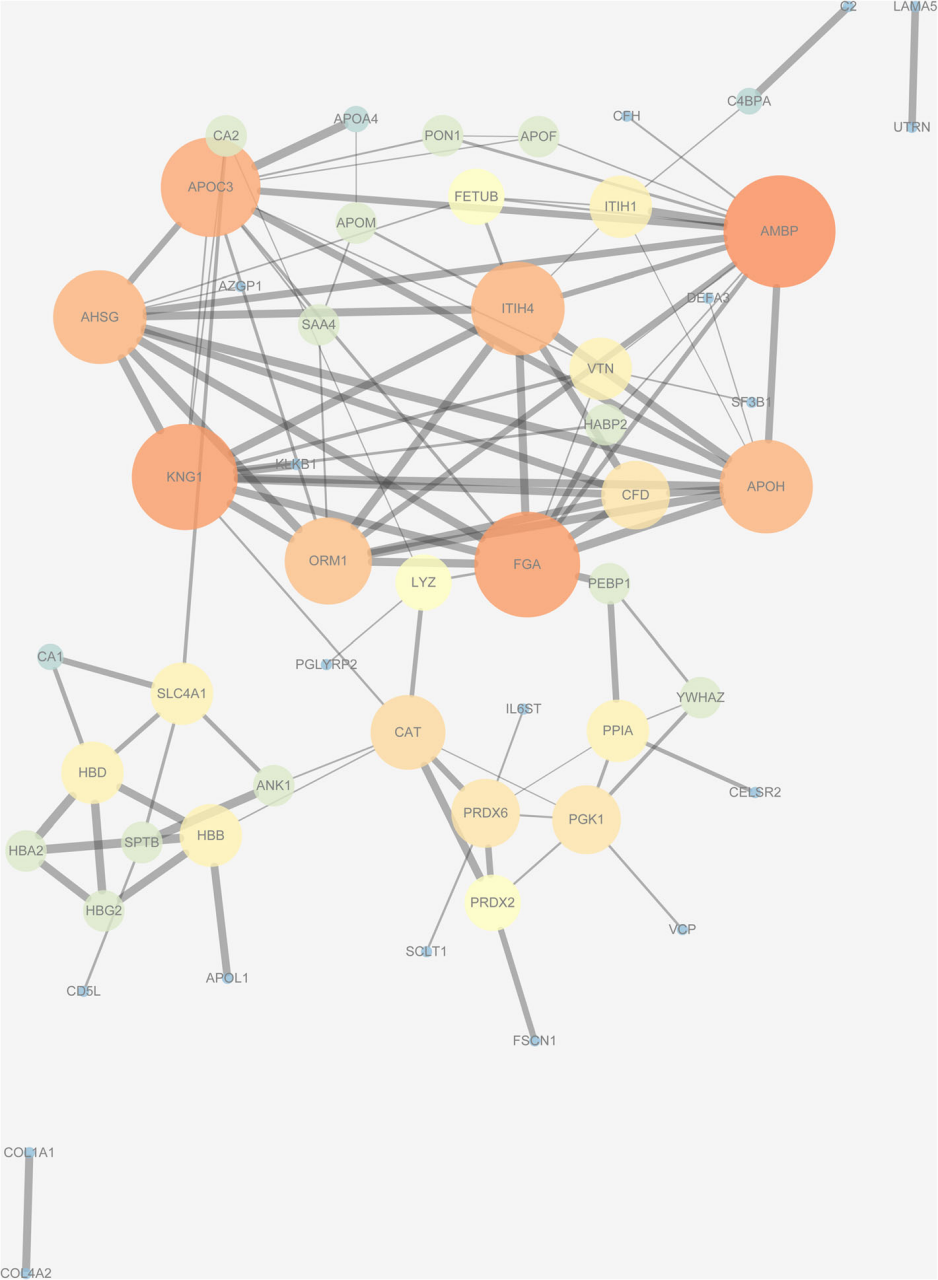
Protein–protein interaction analysis conducted by the String database and Cytoscape software. The most significant 8 proteins related to PDR is displayed

**Fig. 9:**
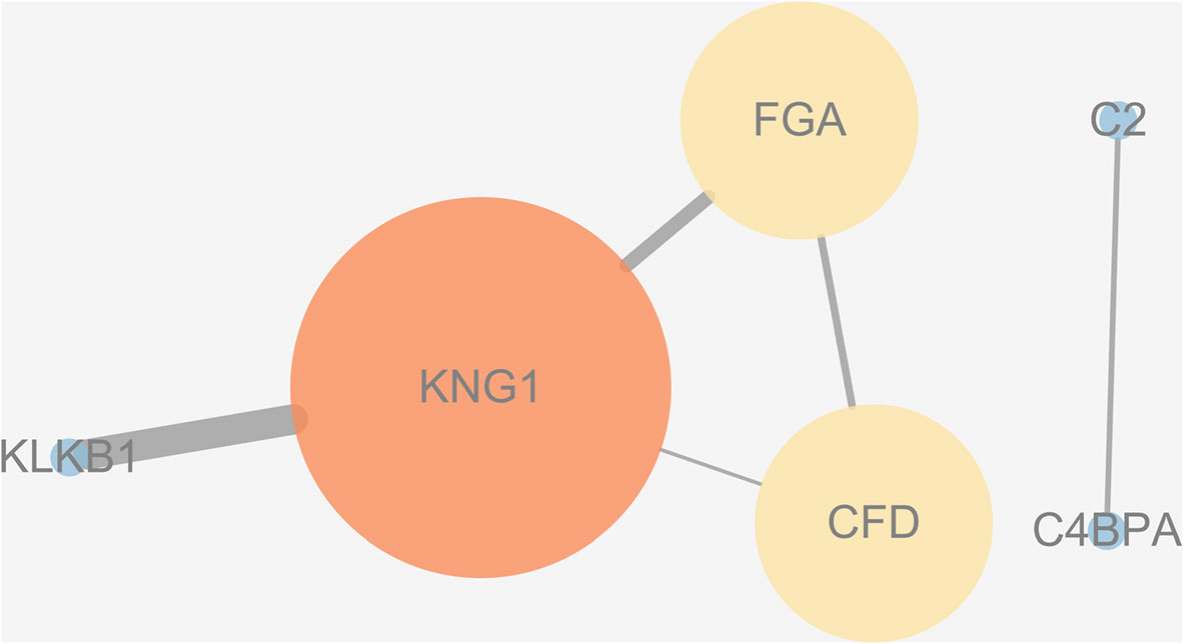
The exclusively involved KEGG pathway and 7 proteins detected by the String database. Complement factor H is an isolated one which has no interaction with other proteins. Complement factor D (CFD) and C4b-binding protein alpha chain interacts mutually and exclusively. Furthers, kallikrein B1 interplays highly with Kininogen-1 and Kininogen-1 has interaction with Fibrinogen alpha chain (FGA) and CFD. Meanwhile, FGA and CFD also interacts

#### Principal component analysis

We conducted principal component analysis on all detected proteins and significant proteins. The separation of the PDR group and the control group demonstrated their difference in protein expression. C5, which contained the most proteins with extreme abundance, was certainly an isolated one in both analyses, as shown in Fig. 3. Moreover, when taking only significant proteins into account, the dots in the same group became closer to each other which stated less difference among the samples. The principal component 1, which indicated that the differences between the two groups can explain the overall analysis results, varied from 53 to 70%, thus illustrating a reduction in confounding factors.

#### Hierarchical clustering analysis

To assess the qualitative and quantitative differences between the PDR and the IMH vitreous proteome, we performed hierarchical clustering of the 18 vitreous samples based on their protein content. The color gradient showed the fold change between the two groups. The quantitative proteomes (*n* = 610) of the PDR and the IMH samples differed and resulted in clear separation of the two disease states (Fig. 4a). When hierarchical clustering was performed on significant proteins only (*n* = 62), the separation became more apparent (Fig. 4b).

### Bioinformatics results

We combined Group A and Group C into Group 1 (*n* = 116) and Group B and Group D into Group 2 (*n* = 222) and carried out bioinformatic analyses such as protein function, related disease, and GO on these two groups. When comparing the two groups, we defined the fold change, which had a significant change of over 2.

#### Protein function

We compared protein functions between the two groups. In Group 1, 111 proteins out of 116 were classified into 50 categories, while in Group 2, 214 proteins were categorized for 64 protein functions.

Some elementary physiological functions, which included polymorphism, signal, glycoprotein and disulfide bond, were of high abundance in both groups, yet they were related to no less than 50% proteins in each group. However, although secreted was associated with over 50% proteins in Group 1, the related proteins in Group 2 was only 30.84%.

Moreover, though immunity was related to 15.32% proteins in Group 1, only 7.48% proteins in Group 2 were relevant.

Among the functions that were related to over 10% of proteins (Fig. 5), transport was identified only in Group 1, while more functions were unique to Group 2 such as membrane, transmembrane helix, transmembrane, cell membrane, calcium, transport, lysosome, methylation and endoplasmic reticulum.

#### Related disease

Related diseases of the significant proteins (Group 1 and Group 2) were analyzed. In Group 1, 87 proteins out of 116 were classified, and in Group 2, 164 out of 222 were identified.

Disease classification was conducted on both groups (Fig. 6). The top 6 disease categories were cardiovascular, metabolic, pharmacogenomic, cancer, neurological and immune. Apart from immune, the other 5 classifications were associated with more proteins in Group 1 than Group 2, yet the fold changes were lower than 2.

Vision-related proteins were the key points of this study. As shown in Table 1, there was no overlap on the proteins associated with vision from the two groups. In Group 1, the relevant proteins included: apolipoprotein F (APOF), carbonic anhydrase 1 (CA1), catalase (CAT), collagen type I alpha 1 chain (COL1A1), complement C2 (C2), complement component 4 binding protein alpha (C4BPA), complement factor D (CFD), complement factor H related 3 (CFHR3), complement factor H (CFH), fibrinogen alpha chain (FGA), paraoxonase 1 (PON1) and vitronectin (VTN). These proteins should be carefully analyzed.

**Table 1:** Vision-related proteins in two groups

Group 1		Group 2	
ID	Name	ID	Name
Q13790	apolipoprotein F (APOF)	P35625	TIMP metallopeptidase inhibitor 3 (TIMP3)
P00915	carbonic anhydrase 1 (CA1)	Q96JP9	cadherin related family member 1 (CDHR1)
P04040	Catalase (CAT)	P00488	coagulation factor XIII A chain (F13A1)
P02452	collagen type I alpha 1 chain (COL1A1)	P02458	collagen type II alpha 1 chain (COL2A1)
P06681	complement C2 (C2)	P05813	crystallin beta A1 (CRYBA1)
P04003	complement component 4 binding protein alpha (C4BPA)	P07099	epoxide hydrolase 1 (EPHX1)
P00746	complement factor D (CFD)	P14210	hepatocyte growth factor (HGF)
A0A087WYK9	complement factor H related 3 (CFHR3)	P11215	integrin subunit alpha M (ITGAM)
P08603	complement factor H (CFH)	D3DSM0	integrin subunit beta 2 (ITGB2)
P02671	fibrinogen alpha chain (FGA)	P05362	intercellular adhesion molecule 1 (ICAM1)
P27169	paraoxonase 1 (PON1)	P30492	major histocompatibility complex, class I, B (HLA-B)
P04004	Vitronectin (VTN)	Q5Y7A7	major histocompatibility complex, class II, DR beta 1 (HLA-DRB1)
		P35579	myosin heavy chain 9 (MYH9)
		K7EKW2	olfactomedin 2 (OLFM2)
		Q6UX71	plexin domain containing 2 (PLXDC2)
		O15537	retinoschisin 1 (RS1)
		G8JLJ2	superoxide dismutase 2, mitochondrial (SOD2)
		C9JXZ5	vesicle associated membrane protein 8 (VAMP8)

The two categories that were unique to Group 2 were renal and developmental.

#### GO

GO is a major bioinformatics initiative to unify the representation of gene and gene product attributes across all species [21], which include three categories: biological processes, cellular components and molecular function. Significant proteins were analyzed to get a functional overview. Among all of the significant proteins, 115 in Group 1 and 214 in Group 2 could be recognized by DAVID Bioinformatics Resources through an identifier named UNIPROT ACCESSION.

### Biological processes

In Group 1, 102 proteins were identified for biological processes, and a total of 79 processes were identified while in Group 2, the number of related proteins was 204 which were associated with 99 biological processes. We made a comparison of biological processes that involved over 5% of the proteins in each group (Fig. 7a).

As shown in Fig. 7a, a total of 5 biological processes were related to significantly more proteins (fold change > 2) in Group 1, including receptor-mediated endocytosis, innate immune response, complement activation, classical pathway, complement activation, and Fc-epsilon receptor signaling pathway.

Moreover, 7 biological processes were unique to Group 1, which included negative regulation of endopeptidase activity, immune response, cellular oxidant detoxification, cell-cell adhesion, negative regulation of apoptotic process, hydrogen peroxide catabolic process and inflammatory response. In addition, one biological process could be identified in Group 2 only.

### Cellular component

A total of 105 proteins related to 29 different cellular components in Group 1 could be detected, and 209 proteins from Group 2 were relevant to 59 cellular components. The cellular components that were involved with over 5% proteins were compared. (Fig. 7b).

Extracellular exosome was involved with the most proteins in both groups, and their difference was not significant (fold change < 2). Three cellular components were related to significantly more proteins in Group 1: extracellular space, extracellular region and blood microparticle. Furthermore, 1 cellular component had significantly more proteins in Group 2 which was endoplasmic reticulum.

Lastly, 3 cellular components could be identified in Group 1 only: cytosol, high-density lipoprotein particle and very-low-density lipoprotein particle, while 14 cellular components were unique to Group 2.

### Molecular functioning

In Group 1, 34 different molecular functions were identified, involving 99 proteins in total, while in group 2, 200 proteins were detected that were related to 46 molecular functions. Similar analysis was conducted on molecular functions, which concerned approximately 5% proteins.

Apart from molecular functions that could be detected only in Group 1 or 2, the remaining 6 were all associated with more proteins in Group 1 than 2. However, only 1 out of them owned a fold change over 2: antigen binding.

In addition, the four molecular functions that were unique to Group 1 involved serine-type endopeptidase inhibitor activity, heme binding, phospholipid binding and lipid binding. Furthermore, the two unique to Group 2 were calcium ion binding and GTP binding.

### Protein–protein interaction analysis

The research pertaining to protein–protein interaction was conducted only on Group 1 using the String online database and Cytoscape software [22].

Through the String database, 69 proteins out of 116 were filtered into the PPI network complex, containing 69 nodes and 107 edges, and the remaining 47 did not fall into the PPI network. We processed these statistics using the Cytoscape software and produced an image (Fig. 8). The style of the figure was generated from statistics, to be specific, the size and color were influenced by the degree and the combined score dictated the edge size. It was designed so that low value led to small sizes and dark colors. As shown in the image, the most significant 8 proteins were Protein AMBP (AMBP), Apolipoprotein C-III (APOC3), Kininogen-1 (KNG1), Fibrinogen alpha chain (FGA), Inter-alpha-trypsin inhibitor heavy chain H4 (ITIH4), Alpha-2-HS-glycoprotein (AHSG), Beta-2-glycoprotein 1 (apolipoprotein H) (APOH) and Alpha-1-acid glycoprotein 1 (orosomucoid 1) (ORM1).

### KEGG pathway

When analyzing Group 1 using the String database, it was concluded that the only involved KEGG pathway were complement and coagulation cascades, which referred to 7 proteins: KNG1, FGA, C2, C4b-binding protein alpha chain (C4BPA), CFD, CFH, and kallikrein B1 (KLKB1). Among them, CFH was an isolated one which had no interaction with other proteins. In total, we obtained 7 nodes and 5 edges and presented the data to the Cytoscape software and then generated the figure style from the statistics as mentioned above (Fig. 9). As shown in Fig. 9, CFD and C4BPA interact only with each other. KLKB1 strongly interplayed with KNG1, which interacted with FGA and CFD; FGA and CFD also interacted.

## Discussion

Hitherto, there have been several proteomics studies conducted on DR using serum [23–25], tear fluid [26–28], aqueous humor [29] and vitreous [30, 31] from patients. However, only a few have quantitatively analyzed PDR [32]. Loukovaara S et al. analyzed 138 vitreous humor samples from patients with NPDR or PDR which had been the most extensive diabetic vitreous proteome analysis so far [20]. Gao BB et al. studied characterization of the vitreous proteome in diabetes without diabetic retinopathy and PDR [33]. However, these two studies both compared PDR and patients who were in a diabetic condition, and thus could not reveal the comprehensive understanding of the underlying molecular pathogenesis of PDR.

García-Ramírez M et al. compared proteomic analysis on vitreous humor from type 1 diabetic patients with PDR and from non-diabetic patients with IMH using fluorescence-based difference gel electrophoresis (DIGE) [32]. Our study was carried out on type 2 diabetic patients with PDR and on IMH subjects through MS-based label-free quantitative proteomics analysis. In this study, the proteomic analysis of PDR vitreous was optimized in the following ways. First, strict exclusion criteria were applied to protect our result from interference, such as infection, hemorrhage or opacities. Second, our proteomic analysis was quantitative, which could obtain the exact abundance of the proteins detected in the samples so that the difference of protein expression in these two diseases could be quantified. An analysis of protein distribution was carried out so that we could gain a brief understanding of protein content, sample reliability as well as a clear separation of the two disease states. Detailed bioinformatic analysis was conducted, which included protein function, related disease, biological processes, molecular functioning, cellular components and the KEGG pathway. Therefore, many inspirations about the target protein function, biomarkers, physiological and pathological changes and the related pathways were revealed through this study.

To our knowledge, this research was the first quantitative proteomics analysis to study the protein distribution which could obtain a brief understanding of protein content and sample reliability. A clear separation of the protein expression in the two diseases was observed. When only taking significant proteins into account, the separation became more apparent and the difference among the samples in one group became less because confounding factors were reduced. Besides, more proteins in the control group had abnormal abundance and C5 was the most unreliable one, the reason of which could be that IMH was another disease condition which might be the result of a variety of unknown factors. Due to its complexity, we just counted IMH samples as controls but did not analyze the related proteins or statistics. Moreover, although there was a weaker correlation among IMH samples, a wider protein distribution in PDR samples was detected which illustrated that PDR was a complex disease which influenced a great deal of protein expression.

A comprehensive bioinformatics research was carried out which provided us with a better understanding of the pathomechanism of PDR as well as the molecular signaling pathway, thus could be of great value to the development of therapeutic method. Apolipoproteins have been reported involving with the pathology of PDR [34, 35], here we identified a total of 6 apolipoproteins, 5 were the first time to be found related to PDR while the remaining one, apolipoprotein A-IV (APOA4) was found reduced in the PDR patients compared with the macular hole patients [36] which had conflicts with our result. Besides, several researches related to PDR have been carried out on vision related proteins, such as CA1 [37], CAT [38], CFH [39], PON1 [40] and VTN [41]. However, no researches have been conducted on APOF, COL1A1, C2, C4BPA, CFD and CFHR3, FGA. Furthermore, KNG1 has never been studied in the pathology of PDR. It initiates the kallikrein-kinin system which is related to the blood pressure systems and inflammation, the latter one is known to cause dilation of blood vessels and increase vascular permeability.

However, there are some limitations in our study. First, the IMH patients were enrolled as controls. Although IMH has no protopathy, it does not equal to normal condition. Because any pathological state may lead to changes in protein profile, normal eyes are the best options for the control group, whereas it is against the ethic. Second, further validation study like dot blot, western blot, enzyme-linked immunosorbent assays or immunohistochemistry should have been conducted in order to confirm some proteins of particular interest. Furthermore, the role of a protein in pathogenesis could have been tested by injection of purified protein into an animal model or by using a pharmaceutical invention.

## Conclusions

In conclusion, we identified and quantified 610 proteins in total, which included 338 significant proteins in our study. Protein distribution analysis demonstrated a clear separation of protein expression in PDR and IMH. The protein function analysis illustrated that immunity and transport related proteins might be associated with PDR. In addition, 12 proteins were identified that were related to vision diseases, some of which have already been studied, but more studies should be carried out to analyze the relation between these proteins and PDR as well as the underlying mechanisms. Meanwhile, GO annotation indicated that the proteins in the vitreous may reflected the physiological and pathological changes in retinal lesion. Also, PPI analysis in our study identified the most significant 8 proteins related to PDR. Furthermore, pathway research indicated the complement and coagulation cascades might be the most important pathway and KNG1 is the key protein in the pathology of PDR.

## Abbreviations

AHSG: Alpha-2-HS-glycoprotein
AMD: Age-related macular degeneration
APOA4: apolipoprotein A-IV
APOC3: Apolipoprotein C-III
APOF: Apolipoprotein
APOH: Apolipoprotein H
BCA: Bicinchoninic acid
C2: Complement C2
C4BPA: C4b-binding protein alpha chain
C4BPA: Complement component 4 binding protein alpha
CA1: Carbonic anhydrase 1
CAT: Catalase
CFD: Complement factor D
CFH: Complement factor H
CFHR3: Complement factor H related 3
COL1A1: Collagen type I alpha 1 chain
DIGE: Difference gel electrophoresis
DR: Diabetic retinopathy
FGA: Fibrinogen alpha chain
GO: Gene ontology
HCA: Hierarchical clustering analysis
IMH: Idiopathic macular hole
IRMA: Intraretinal microvascular abnormalities
ITIH4: Inter-alpha-trypsin inhibitor heavy chain H4
KEGG: Kyoto Encyclopedia of Genes and Genomes
KLKB1: Kallikrein B1
KNG1: Kininogen-1
MS: Mass spectrometry
NPDR: Nonproliferative diabetic retinopathy
ORM1: Orosomucoid 1
PC: Principal component analysis
PDR: Proliferative diabetic retinopathy
PON1: Paraoxonase 1
PPI: Protein-protein interaction
VTN: Vitronectin
